# Triptonide effectively inhibits triple-negative breast cancer metastasis through concurrent degradation of Twist1 and Notch1 oncoproteins

**DOI:** 10.1186/s13058-021-01488-7

**Published:** 2021-12-18

**Authors:** Mengli Zhang, Mei Meng, Yuxi Liu, Jindan Qi, Zhe Zhao, Yingnan Qiao, Yanxing Hu, Wei Lu, Zhou Zhou, Peng Xu, Quansheng Zhou

**Affiliations:** 1 Cyrus Tang Hematology Center, Jiangsu Institute of Hematology, Key Laboratory of Thrombosis and Hemostasis, Ministry of Health, 2011 Collaborative Innovation Center of Hematology, Soochow University, 199 Ren Ai Road, Suzhou Industrial Park, Suzhou, 215123 Jiangsu People’s Republic of China; 2 State Key Laboratory of Radiation Medicine and Protection, School of Radiation Medicine and Protection, Soochow University, Suzhou, 215123 Jiangsu People’s Republic of China; 3 National Clinical Research Center for Hematologic Diseases, The First Affiliated Hospital of Soochow University, Suzhou, 215006 Jiangsu People’s Republic of China; 4 School of Nursing, Soochow University, Suzhou, 215006 Jiangsu People’s Republic of China

**Keywords:** Breast cancer, Metastasis, Triptonide, Twist1, Notch1

## Abstract

**Background:**

Triple-negative breast cancer (TNBC) is highly metastatic and lethal. Due to a lack of druggable targets for this disease, there are no effective therapies in the clinic.

**Methods:**

We used TNBC cells and xenografted mice as models to explore triptonide-mediated inhibition of TNBC metastasis and tumor growth. Colony formation assay was used to quantify the tumorigenesis of TNBC cells. Wound-healing and cell trans-well assays were utilized to measure cell migration and invasion. Tube formation assay was applied to access tumor cell-mediated vasculogenic mimicry. Western blot, quantitative-PCR, immunofluorescence imaging, and immunohistochemical staining were used to measure the expression levels of various tumorigenic genes in TNBC cells.

**Results:**

Here, we showed that triptonide, a small molecule from the traditional Chinese medicinal herb *Tripterygium wilfordii* Hook F, potently inhibited TNBC cell migration, invasion, and vasculogenic mimicry, and effectively suppressed TNBC tumor growth and lung metastasis in xenografted mice with no observable toxicity. Molecular mechanistic studies revealed that triptonide strongly triggered the degradation of master epithelial-mesenchymal transition (EMT)-inducing protein Twist1 through the lysosomal system and reduced Notch1 expression and NF-κB phosphorylation, which consequently diminished the expression of pro-metastatic and angiogenic genes *N-cadherin*, *VE-cadherin*, and vascular endothelial cell growth factor receptor 2 (*VEGFR2*).

**Conclusions:**

Triptonide effectively suppressed TNBC cell tumorigenesis, vasculogenic mimicry, and strongly inhibited the metastasis of TNBC via degradation of Twist1 and Notch1 oncoproteins, downregulation of metastatic and angiogenic gene expression, and reduction of NF-κB signaling pathway. Our findings provide a new strategy for treating highly lethal TNBC and offer a potential new drug candidate for combatting this aggressive disease.

**Supplementary Information:**

The online version contains supplementary material available at 10.1186/s13058-021-01488-7.

## Highlights

1. Triptonide effectively suppresses TNBC tumor growth and metastasis in xenografted mice.
2. The migration and angiogenesis of TNBC cells are markedly inhibited by triptonide.
3. Triptonide triggers the degradation of oncoproteins Twist1 and Notch1 in TNBC cells.
4. Our findings provide a new strategy for treating aggressive and metastatic TNBC.

## Introduction

Triple-negative breast cancer (TNBC) is characterized by the absence of estrogen receptors (ER), progesterone receptors (PR), and human epidermal growth factor receptor 2 (HER2) and is highly metastatic and lethal [1, 2]. Although substantial efforts have gone into identifying treatments for TNBC in recent decades, the median survival time of TNBC patients is still less than 15 months largely due to the absence of effective drugs in the clinic [3, 4]. Since its discovery, TNBC has been a challenge in the cancer treatment field [3–5], warranting the discovery of novel, potent therapeutics against TNBC.

TNBC has multiple genetic and epigenetic abnormalities. It was recently reported that 411 genes were overexpressed in TNBC tumor tissue compared to normal breast tissue and that multiple tumorigenic and metastatic signaling pathways were aberrantly activated [6, 7]. TNBC cells possess high migratory and invasive abilities and easily disseminate from the primary tumor site to multiple distant organs, such as lung, bone, liver, and brain, resulting in organ dysfunction and patient death [8–10].

Epithelial–mesenchymal transition (EMT) seems to play a pivotal role in TNBC initiation, metastasis, and drug resistance [9, 11, 12]. Numerous studies have demonstrated that aberrant overexpression of EMT-inducing genes, such as *Twist1, Snail1, Snail2, Zeb1, Zeb2, N-cadherin*, and the master stemness gene *Notch1*, triggers cell de-differentiation and cancer stem cell genesis [8, 9, 11]. Particularly, concurrent overexpression of the oncogenic genes *Twist1, Snail1,* and *Notch1* strongly induces EMT and enhances stemness, thereby inducing cancer and metastasis [9, 11–14].

*Twist1* is a master EMT-inducing gene and plays a critical role in cancer metastasis in various malignant tumors [7]. *Twist1* overexpression promotes TNBC metastasis and is associated with poor prognosis of TNBC patients [13, 15]. Accordingly, Twist1 has been a target for the development of therapeutics against TNBC [16–19]. Several small molecules, such as thymoquinone [16] and tamoxifen [17], reduce *Twist1* expression in cell and animal models and inhibit TNBC cell migration and invasion in vitro; however, these agents exert only moderate efficacy and have not yet entered clinical trials. Therefore, potent Twist1-targeted therapeutics remain to be explored.

*Notch1* is an essential gene for cancer stem cell genesis and is a hallmark of TNBC [20–23]. Overexpression of *Notch1* is closely associated with a poor prognosis in TNBC patients [24]. Accordingly, Notch1 suppression is a potential strategy for cancer therapy [25–29]. However, Notch1-targeted drugs against TNBC have not advanced to clinical trials due to low anti-cancer efficacy in preclinical studies [23]. Thus, there is a critical need for effective Notch1-targeted therapeutics for TNBC. Since multiple oncogenic genes and signal pathways contribute to the progression of TNBC, targeting a single *Notch 1* gene appears to run short of ways in dealing with the situation of TNBC progression, suggesting that concurrently targeting multiple tumorigenic genes and signaling pathways are sensible strategy for TNBC therapy.

TNBC cells gain high tumorigenic and metastatic capabilities via gene mutation and also exhibit aberrant gene overexpression. Because multiple oncogenic genes are simultaneously overexpressed in TBNC [6–9], targeting a single gene is typically insufficient to treat the disease. Therefore, we hypothesized that concurrently targeting multiple oncogenic genes, such as *Twist1* and *Notch1*, may increase anti-TNBC efficacy, and we explored active components with potential anti-TNBC effects from the traditional Chinese medicinal herbs. Our results showed that triptonide (MW:358 Da), a small molecule from the traditional Chinese medicinal herb *Tripterygium wilfordii* Hook F [30, 31], exerted potent anti-TNBC effects.

It was recently reported that triptonide exerts strong inhibitory effects on acute myeloid leukemia, lymphoma, lung cancer, gastric cancer, and pancreatic cancer via reducing cancer cell stemness and oncogenic signaling pathways, and suppressing cancer cell tumorigenesis and vasculogenic mimicry [32–38]. More recently, Gao et al. reported that triptonide inhibits TNBC cell tumorigenesis via downregulation of several cancer stem cell-associated genes, up-regulation of Snail1 expression, and induction of a Snail1-associated feedback mechanism for triptonide resistance [39]. Of note, it is well known that Smail1 is aberrantly overexpressed in various types of malignant tumors including TNBC [40–43], overexpression of Snail triggers EMT [42, 44–47], carcinogenesis [48–51], and drug resistance [52, 53], and is closely associated with poor prognosis in cancer patients [40, 43, 46, 47, 50, 51]. Accordingly, inhibition of Snail1 in cancer has become a new strategy for developing cancer therapeutics [52–54]. Therefore, the effect of triptonide on Snail expression in TNBC cells remains to be verified, and the mechanisms underlying triptonide-mediated anti-TNBC need to be elucidated.

In the current investigation, we found that triptonide potently suppressed TNBC cell migration, invasion, and angiogenesis in vitro and effectively suppressed tumor growth and metastasis in xenografted mice. This effect was mediated by triptonide-induced Twist1 and Notch1 protein degradation, which consequently inhibited downstream NF-κB signaling, suppressing the expression of angiogenic and metastatic genes *VE-cadherin*, *VEGFR2*, and *N-cadherin*. Of note, triptonide does not increase Snail1 expression in TNBC cell lines we tested. Our findings provide a new strategy for concurrently inhibition of multiple oncogenic genes and tumorigenic signaling pathways in TNBC and offer a novel drug candidate against metastatic TNBC.

## Methods

### Materials

Triptonide was from Chengdu Must Biotech Ltd. with a purity > 98%. MG132, cycloheximide, and chloroquine diphosphate were from MCE (Shanghai, China). Revert Aid TM First Strand cDNA Synthesis Kit was from Fermentas Life Sciences (Walsham, Massachusetts). Rabbit monoclonal antibody to Notch1 and other signaling proteins were from Cell Signaling Technology (Beverly, MA). Mouse and Rabbit polyclonal antibodies to Twist1 were from ABCAM (Cambridge, UK). Breast cancer cell lines MDA-MB-231, MDA-MB-468, and BT-549 were from ATCC (Rockefeller, Maryland).

### Cell culture

TNBC cell lines MDA-MB-231, MDA-MB-468, and BT-549 were cultured in Dulbecco's modified Eagle medium (DMEM) supplemented with 10% fetal bovine serum (complete medium). The cells were maintained at 37 °C in a humidified atmosphere of 5% CO_2_ as we described previously [32].

### Tumor xenograft mice and triptonide treatment

The tumor xenograft mice were conducted in accordance with protocols approved by the Institutional Animal Care and Use Committee (IACUC) of Soochow University as previously reported [35, 38]. In brief, 10^7^ MDA-MB-231 cells were injected into the breast of NOD-SCID female mice (18–22 g) and the mice were randomly divided into two groups (triptonide treatment group and control group, 5 mice/group). After 10 days when the tumors grew up, triptonide in saline at a dose of 3 mg/kg or saline as a control was intraperitoneally injected daily for 95 days. Tumor size was measured and tumor volume was calculated according to the formula: tumor volume = 0.55 × length × width^2^. After 95 days of triptonide treatment, the mice were sacrificed. The lungs and other important organs were isolated, weighed, washed with PBS, fixed with formaldehyde, sectioned, and analyzed by hematoxylin–eosin (H&E) staining.

### Immunofluorescence microscopy

MDA-MB-231 and MDA-MB-468 cells were first seeded onto glass cover slips for 24 h, then treated with triptonide at the concentrations of 0–10 nM for 72 h. The cells were fixed with 4% paraformaldehyde and incubated with primary antibody against Twist1 at 4 °C overnight, followed by incubation with secondary antibodies, counterstained with 4, 6-diamidino-2-phenylindole (DAPI), and subjected to be imaged under confocal microscopy [36].

### Immunohistochemical staining

The lung tumor tissues from xenografted mice were sectioned, and the slides were stained with anti-Twist1 primary antibody at 4 °C overnight, followed by incubation with HRP-labeled goat anti-rabbit secondary antibody for 1 h at room temperature, and stained with 3,3-diaminobenzidine (DAB) solution. The slides were imaged using Leica microscope.

### Cell growth analysis

MDA-MB-231 and MDA-MB-468 cells were incubated with triptonide at the concentrations of 0–320 nM for 70 h; 10 μL MTT solution was added to each well and incubated at 37 °C for 2 h. The dual fluorescence wavelength absorbance at 560 and 590 nm was measured using SpectraMax M5 reader, respectively. The IC_50_, defined as the drug concentration at which cell growth was inhibited by 50%, was assessed by SPSS 16.0.

### Colony formation assay

MDA-MB-231 and MDA-MB-468 cells were treated with triptonide at the concentrations of 0–10 nM for 72 h; the viable cells were harvested, and counted. The 5000 viable cells together with 0.5% low melting agarose were seeded into the 35 mm dishes and incubated at 37 °C in 5% CO_2_ for 10 days in the absence of triptonide; the number of colonies was counted under a Zoom-Stereo microscope SZX16 as we previously described [55].

### Cell migration assay

MDA-MB-231 and MDA-MB-468 cells were seeded in six-well plates and incubated for 24 h, and then, the cell monolayer was wounded by a plastic tip. The wounded monolayer was then incubated with triptonide at the concentrations of 0–10 nM for 24 h. The migrated tumor cells were stained with Wright-Giemsa solution, imaged under a microscopy using five randomly chosen fields for each well, and statistically analyzed.

### Cell invasion assay

The MDA-MB-231 cells were seeded into the upper chambers of trans-well plate pre-coated with 12.5% Matrigel at a density of 2.0 × 10^5^ cells/mL and incubated without or with triptonide at the concentrations of 0–10 nM. After 24 h, the invaded cells in the lower chamber were fixed, stained with Wright-Giemsa solution, and photographed as we described before [56].

### Tube forming assay

MDA-MB-231 cells were pre-treated with triptonide at the concentrations of 0–10 nM for 72 h and the viable 2 × 10^4^ cells in 0.5 ml DMEM complete medium were transferred to each well of a 24-well plate containing 0.3 mL Matrigel matrix. After incubation at 37 °C, 5% CO_2_ for 8 h, the tubes were fixed and stained with Wright-Giemsa solution, and photographed by OLYMPUS FSX-100 microscope.

### RT-PCR and real-time quantitative PCR

Total RNA was extracted from triptonide-treated cells, and reversely transcribed into cDNA using the RevertAid First Strand cDNA Synthesis Kit. RT-PCR and real-time quantitative PCR (QT-PCR) were performed with SYBRGreen protocol as we previously described [57]. The primers of RT-PCR and QT-PCR are listed in the Additional file 1: Tables S1 and S2, respectively.

### Western blotting

Proteins from MDA-MB-231 and MDA-MB-468 cells were extracted using the M-PER Mammalian Protein Extraction Kit. Equal amounts of protein were loaded onto each lane and resolved by sodium dodecyl sulfate polyacrylamide gel electrophoresis with tris–glycine running buffer, and the proteins were transferred to nitrocellulose membranes. The membranes were incubated with primary antibodies at 4 °C overnight, followed by incubation with HRP-coupled secondary antibody for 1 h at room temperature. Blots were visualized using enhanced chemiluminescence detection reagents and exposed to X-ray film as we reported before [38].

### Statistical analysis

All results represent the mean ± S.E. Differences between the groups were assessed by one-way ANOVA using GraphPad Prism 7.02. Statistical comparisons were performed using the Student's t test, and the significance of differences was indicated as *P < 0.05 and **P < 0.01.

## Results

### Triptonide potently inhibits TNBC cell proliferation and tumor growth

**Fig. 1:**
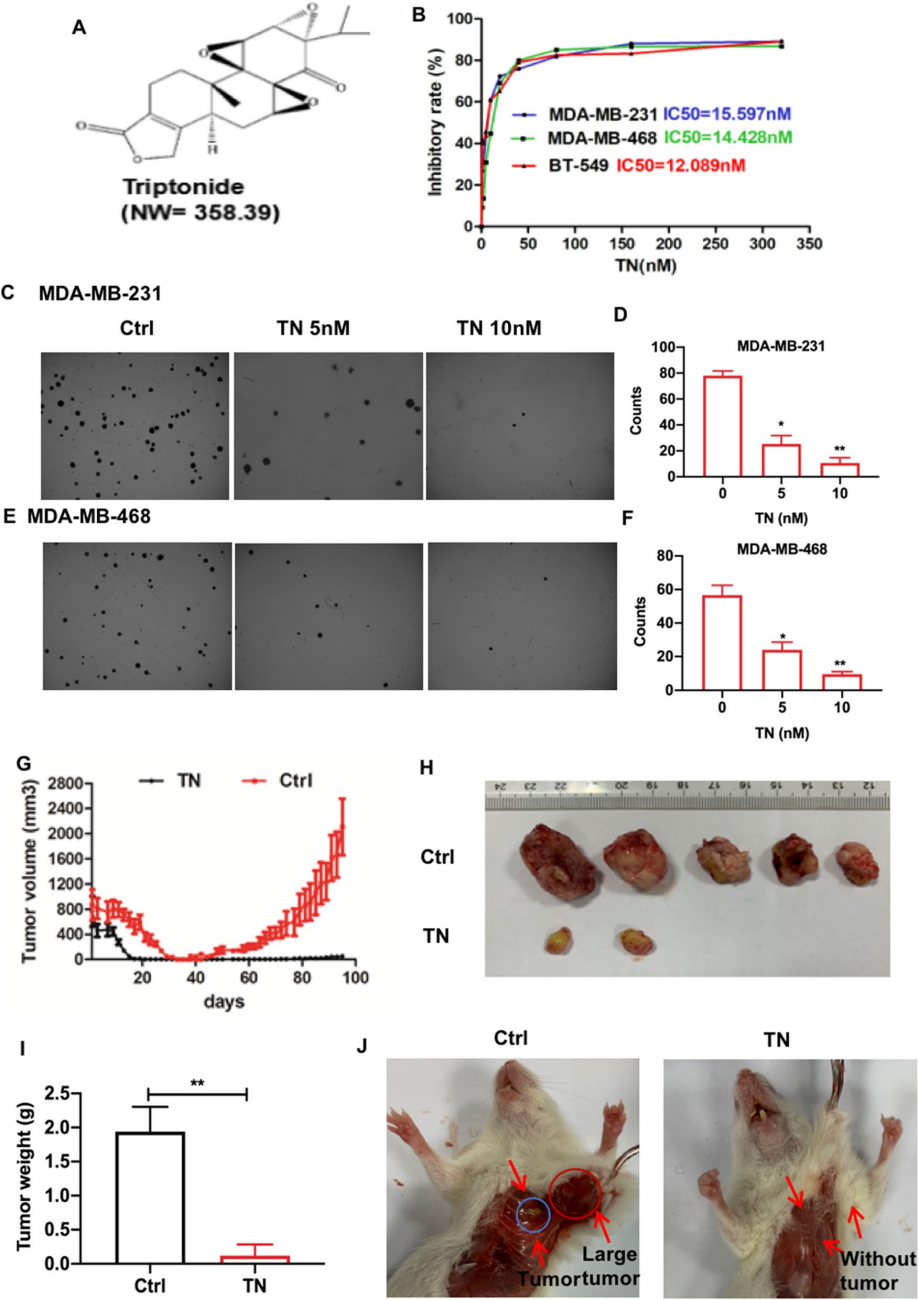
**Triptonide potently inhibits TNBC cell proliferation and tumor growth in xenografted mice.** The triple-negative breast cancer (TNBC) cell lines MDA-MB-231, MDA-MB-468, and BT-549 in the 96-well plate (n-6) were incubated with triptonide (TN, **A**) at the concentrations of 0–320 nM, respectively. Cell growth was detected by MTT assay (**B**). MDA-MB-231 and MDA-MB-468 cells were incubated with TN for 10 days at the concentrations of 0–10 nM; the colony numbers in each dish were imaged (**C**, **E**, 40 ×), counted, and statistically analyzed (**D**, **F**). The results represent three independent experiments. *P < 0.05, **P < 0.01. The five xenografted NOD/ SCID mice/group were treated with TN in saline at the dose of 3 mg/kg or saline as a control once a day. The tumor volume in the mice was recorded every other days (**G**). The tumors were first imaged (**H**), then weighed (**I**). The tumors were apparently bigger in control mice than TN-treated mice (**J**); notably, the tumors spread to the area out of breasts and were visible over the ribs in control xenografted mice, which was indicated by a red circle and an arrow (**J**, left panel), while the blue circle indicates the small tumor in the original transplantation site of the mouse breasts

We first investigated the anti-TNBC effect of triptonide (Fig. 1A) in vitro. Triptonide inhibited the proliferation of all three TNBC cell lines, MDA-MB-231, MDA-MB-468, and BT-549, with IC_50_ values of 15.6, 14.4, and 12.1 nM, respectively (Fig. 1B). Triptonide also markedly reduced the colony numbers of highly metastatic MDA-MB-231 (Fig. 1C, D) and MDA-MB-468 cells (Fig. 1E, F) in a concentration-dependent manner, suggesting that triptonide is a potent inhibitor of TNBC cell tumorigenesis.

We studied the anti-tumor and anti-metastatic effects of triptonide in xenografted mice. TNBC MDA-MB-231 cells together with Matrigel, which was used to mimic the extracellular matrix of the tumor microenvironment, were injected into the breast of NOD-SCID female mice. After 95 days, tumor volume reached 2400 mm^3^ in control mice, but only 100 mm^3^ in triptonide-treated mice (Fig. 1G). In the control group, all five mice had substantial tumor growth. Strikingly, in the triptonide group, three mice exhibited complete remission, while the two remaining mice had significantly reduced tumor growth (Fig. 1H). Similarly, tumor weights were reduced in triptonide-treated mice (reaching a tumor-inhibitory rate of 98%) compared to that of control mice (Fig. 1I). Of note, triptonide did not significantly affect the organ index (organ weight/mouse body weight) of the lung, heart, spleen, kidney, and liver, and no obvious complications were observed. Collectively, these data suggest that triptonide effectively inhibits TNBC tumor growth in xenografted mice without obvious side effects.

### Triptonide effectively inhibits TNBC cell migration and lung metastasis

**Fig. 2:**
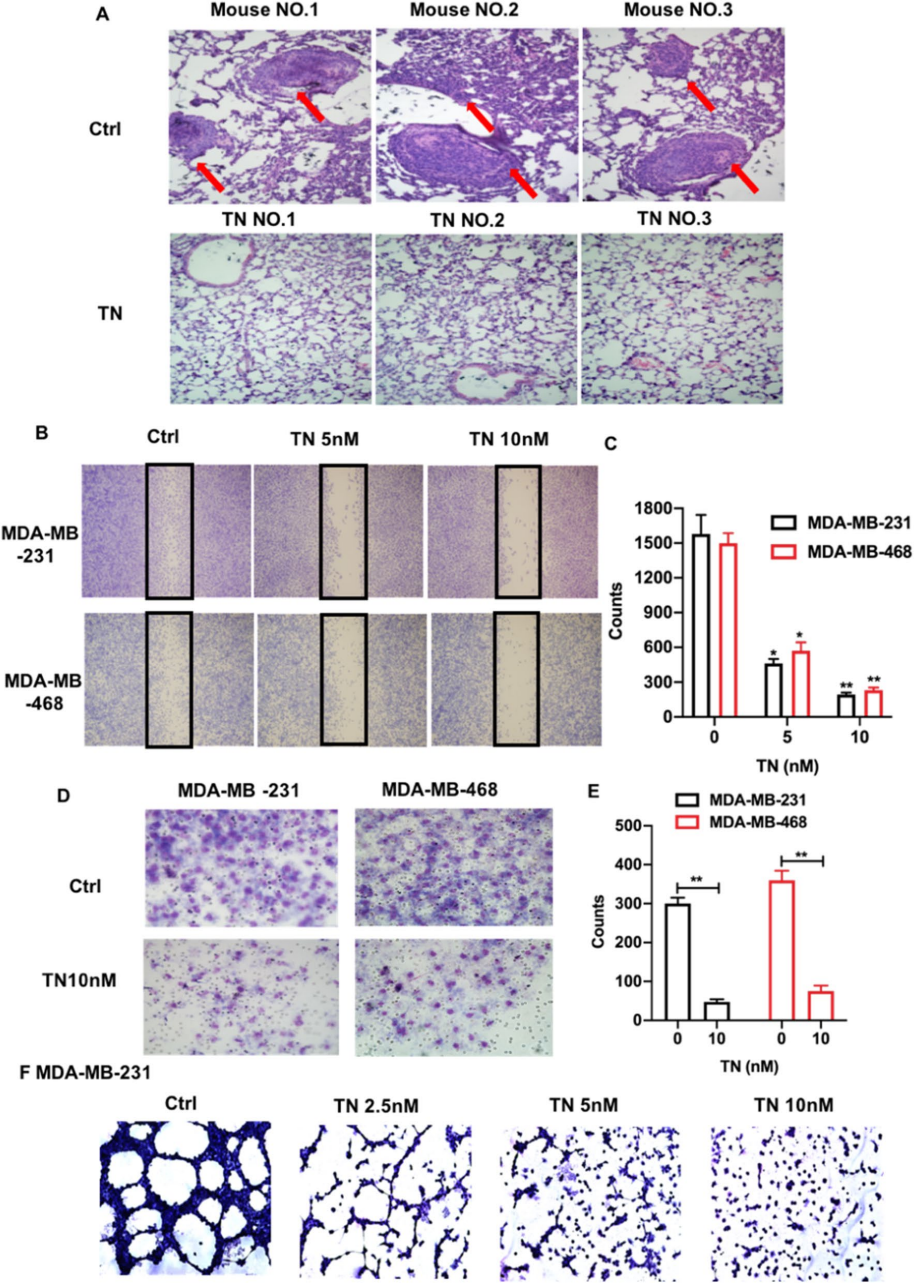
**Triptonide notably inhibits TNBC cell invasion and lung metastasis.** The lungs in triptonide (TN)-treated and control (Ctrl) mice were fixed, subjected to H&E staining, and imaged with a microscope (400 ×). Tumors were appeared in the lung tissues of control mice (**A**, up panels), but were absent in the lung tissues of TN-treated mice (**A**, low panels). The numbers of migrated MDA-MB-231 and MDA-MB-468 cells in the wounded area were first imaged and counted; then, the cell migration-inhibitory rate was calculated (**B**, **C**). Cell invasion was performed by a trans-well system. MDA-MB-231 and MDA-MB-468 cells in the bottom chamber that invaded from the up chamber were fixed, stained with Wright–Giemsa solution, imaged using OLYMPUS CKX31 microscope (**D**), and statistically analyzed (**E**). The tumor cell tube-forming assay showed that TN at the concentration of 10 nM almost completely inhibited MDA-MB-231 cell formation of capillary like structures (**G**). The results represent three independent experiments. *P < 0.05 and **P < 0.01

We investigated the effect of triptonide on TNBC metastasis in xenografted mice. In control mice, the tumors were large and visible over the ribs; however, no tumors were visible at this site in triptonide-treated mice (Fig. 1J), suggesting that triptonide effectively suppressed TNBC dissemination to nearby tissues. This was supported by H&E staining that revealed the presence of tumors in lung tissues of control mice, but not in lung tissues of triptonide-treated mice (Fig. 2A). These data suggest that triptonide effectively inhibits TNBC metastasis from the breast to the lung.

We further investigated the cellular mechanisms underlying triptonide-induced inhibition of TNBC dissemination. In a wound-healing assay, the migration of MDA-MB-231 and MDA-MB-468 cells was notably inhibited by triptonide in a concentration-dependent manner (Fig. 2B, C) with robust inhibition at 10 nM triptonide (Fig. 2D, E). Given that TNBC cells can directly form tumor blood vessels through vascular mimicry, we also examined the effect of triptonide on the ability of TNBC cells to form tube-like structures in vitro. Strikingly, 10 nM triptonide almost completely suppressed tube-like structure formation in MDA-MB-231 cells (Fig. 2F), suggesting that triptonide is a novel, potent inhibitor of TNBC cell vascular mimicry. Collectively, triptonide effectively inhibits TNBC cell migration, invasion, vascular mimicry, and metastasis.

### Triptonide inhibits TNBC cell metastasis by triggering concurrent degradation of Twist1 and Notch1 oncoproteins

**Fig. 3:**
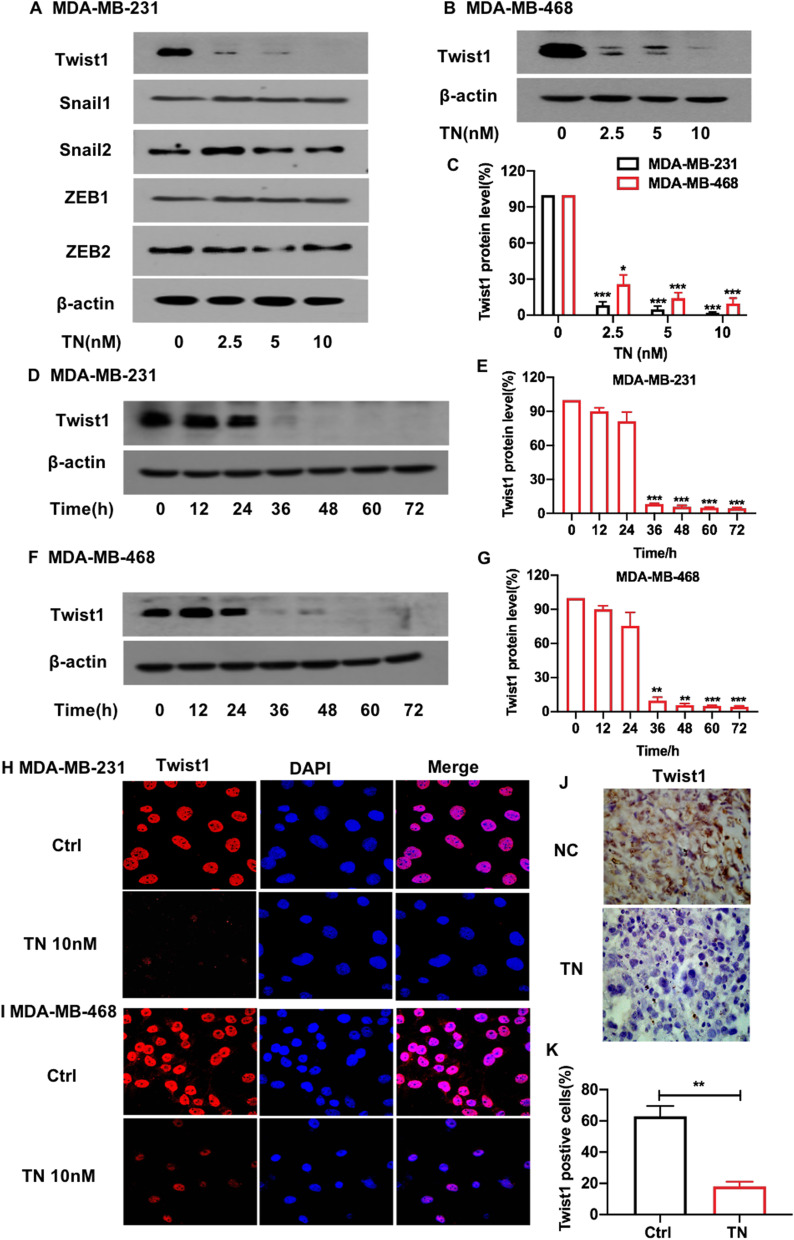
**Triptonide markedly reduces Twist1 protein levels in TNBC cells and in the tumor tissues of xenografted mice. **MDA-MB-231 and MDA-MB-468 cells were treated with triptonide (TN) for 72 h at the concentrations of 0–10 nM. Twist1 protein levels were detected using Western blotting (**A**, **B**), and the bands were scanned and statistically analyzed (**C**). Additionally, the cells were treated with 10 nM TN for 0, 12, 24, 36, 48, and 72 h, respectively, Twist1 protein levels were measured (**D**, **F**) and statistically analyzed (**E**, **G**). Twsist1 protein levels in MDA-MB-231 and MDA-MB-468 cells were also detected using immunofluorescent staining and confocal microscopy (**H**, **I**, × 2400). Furthermore, Twist1 protein levels in the lung tissues of xenografted mice were detected by immunohistochemistry staining (**J**, × 1000) and statistically analyzed (**K**). The results represent three independent experiments. *P < 0 .05, **P < 0.01

We studied the molecular mechanisms underlying triptonide-mediated anti-TNBC cell metastasis. Western blotting showed that triptonide notably decreased the levels of Twist1 in MDA-MB-231 (Fig. 3A, C) and MDA-MB-468 cells (Fig. 3B, C) in a concentration-dependent manner, but the levels of Twist1 mRNA in MDA-MB-231 and MDA-MB-468 cells were not significantly altered in response to triptonide, suggesting that triptonide may reduce Twist1 protein stability in TNBC cells. The protein levels of other EMT genes, such as *Snail1*, *Snail2*, *Zeb1*, and *Zeb2*, were not significantly affected by triptonide (Fig. 3A). Of note, triptonide did not significantly change the levels of Snail1 mRNA and protein in both MDA-MB-231 and MDA-MB-468 cells (Additional file 1: Figure S1),

We analyzed changes in Twist1 protein levels over time following triptonide treatment. Twist1 protein expression was almost completely abolished in MDA-MB-231 (Fig. 3D, E) and MDA-MB-468 cells (Fig. 3F, G) after 72 h of triptonide treatment. To corroborate our findings, immunofluorescent staining and confocal microscopy showed that triptonide markedly decreased the levels of Twist1 protein in MDA-MB-231 (Fig. 3H) and MDA-MB-468 cells (Fig. 3I). Furthermore, immunohistochemical analysis of lung sections from xenografted mice revealed that triptonide significantly reduced the levels of Twist1 protein in metastatic lung tissues compared to control mouse lung tissues (Fig. 3J, K). Therefore, triptonide decreases Twist1 protein levels in TNBC cells.

**Fig. 4:**
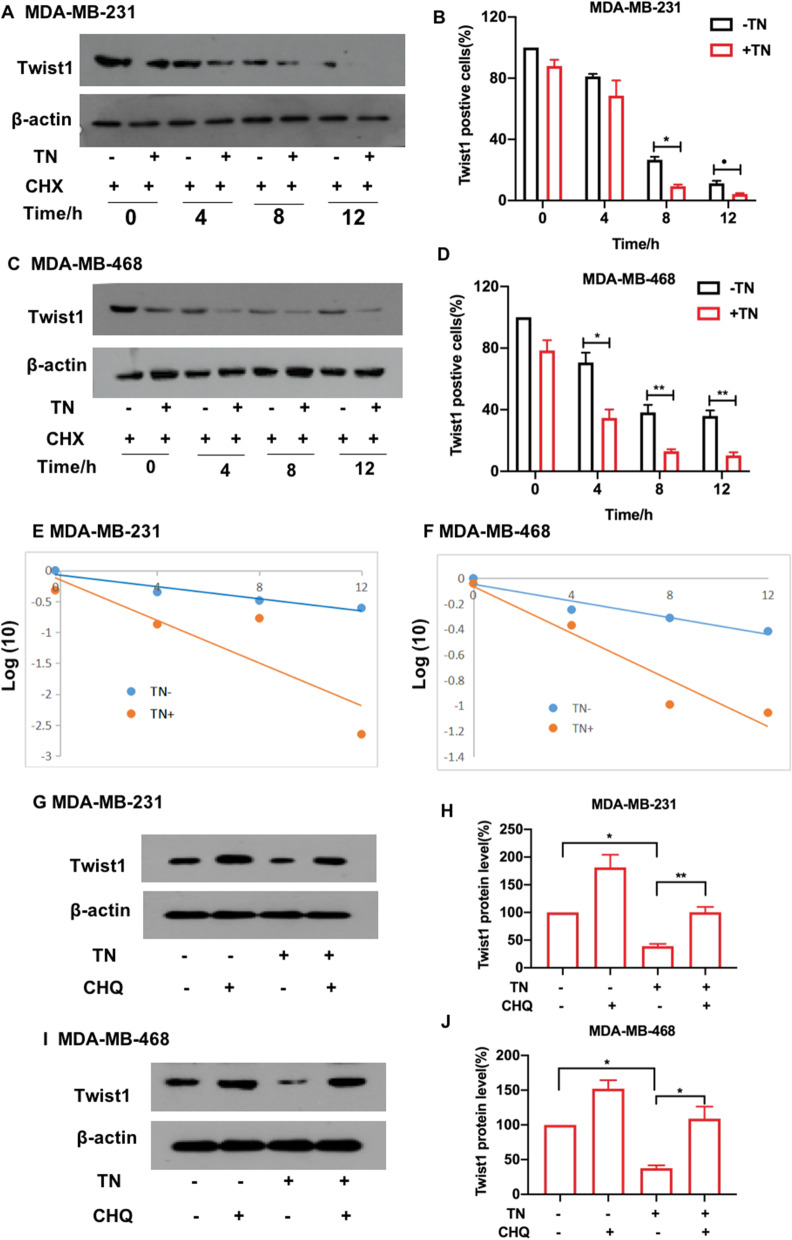
**Triptonide promotes Twist1 degradation in TNBC cells through the lysosomal system. **MDA-MB-231 and MDA-MB-468 cells were first incubated with 10 nM triptonide (TN) for 24 h, followed by incubation with the translational elongation inhibitor cycloheximide (CHX) at the concentration of 20 μM. After CHX treatment for 0 h, 4 h, 8 h, and 12 h, respectively, Twist1 protein levels were measured using Western blotting (**A**, **C**), and the bands were scanned and statistically analyzed (**B**, **D**). Twist1 protein levels were sharply decreased in MDA-MB-231 (**E**) and MDA-MB-468 cells (**F**) in the presence of TN, suggesting that triptonide triggers Twist1 protein degradation in the cells. Additionally, 20 μM chloroquine significantly inhibited triptonide-induced Twist1 protein degradation in MDA-MB-231 (**G**, **H**) and MDA-MB-468 cells (**I**, **J**). The results represent three independent experiments. *P < 0.05, **P < 0.01

Interestingly, we observed that triptonide-induced Twist1 protein decay in TNBC cells was enhanced in the presence of the translational elongation inhibitor cycloheximide (CHX). Twist1 protein levels were reduced in a time-dependent manner after treating cells with 10 nM triptonide together with 20 μM CHX (Fig. 4A–D). Twist1 protein levels decreased faster in triptonide-treated MDA-MB-231 (Fig. 4E) and MDA-MB-468 cells (Fig. 4F) than in control cells without triptonide treatment, suggesting that triptonide promotes Twist1 degradation in TNBC cells.

**Fig. 5:**
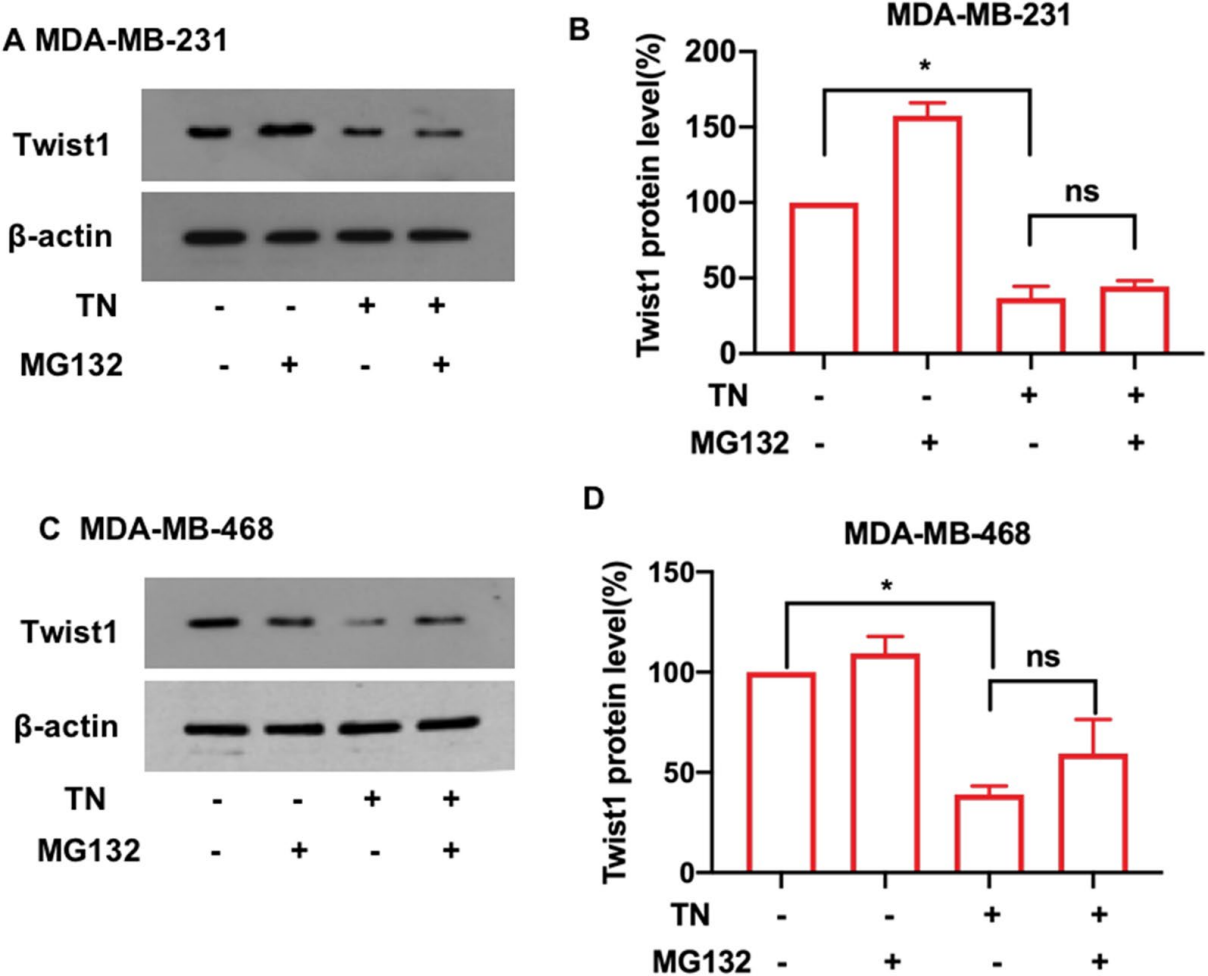
**Triptonide-mediated degradation of Twist1 in TNBC cells was not dependent on the ubiquitin–proteasome system.** After MDA-MB-231 and MDA-MB-468 cells were treated with or without 10 nM triptonide (TN) and with or without 20 μM of the ubiquitin–proteasome inhibitor MG132, Twist1 protein levels were detected by Western blotting (**A**, **C**) and statistically analyzed (**B**, **D**). The results showed that TN-mediated degradation of Twist1 protein in these cells was not significantly changed, suggesting that TN-mediated degradation of Twist1 is not dependent on the ubiquitin–proteasome system. The results represent three independent experiments. *P < 0.05, **P < 0.01

We further investigated whether triptonide-induced Twist1 degradation in TNBC cells occurs through the lysosomal or proteasomal systems. MDA-MB-231 and MDA-MB-468 cells were incubated for 12 h with 10 nM triptonide together with 20 μM of the lysosomal inhibitor chloroquine. Chloroquine significantly inhibited triptonide-triggered degradation of Twist1 in MDA-MB-231 (Fig. 4G, H) and MDA-MB-468 cells (Fig. 4I, J). However, when these cells were incubated for 12 h with 10 nM triptonide together with 20 μM of the proteasome inhibitor MG132, the levels of Twist1 protein were not significantly affected (Fig. 5A–D). Therefore, triptonide-triggered Twist1 protein degradation in TNBC cells occurs mainly through the lysosomal system.

**Fig. 6:**
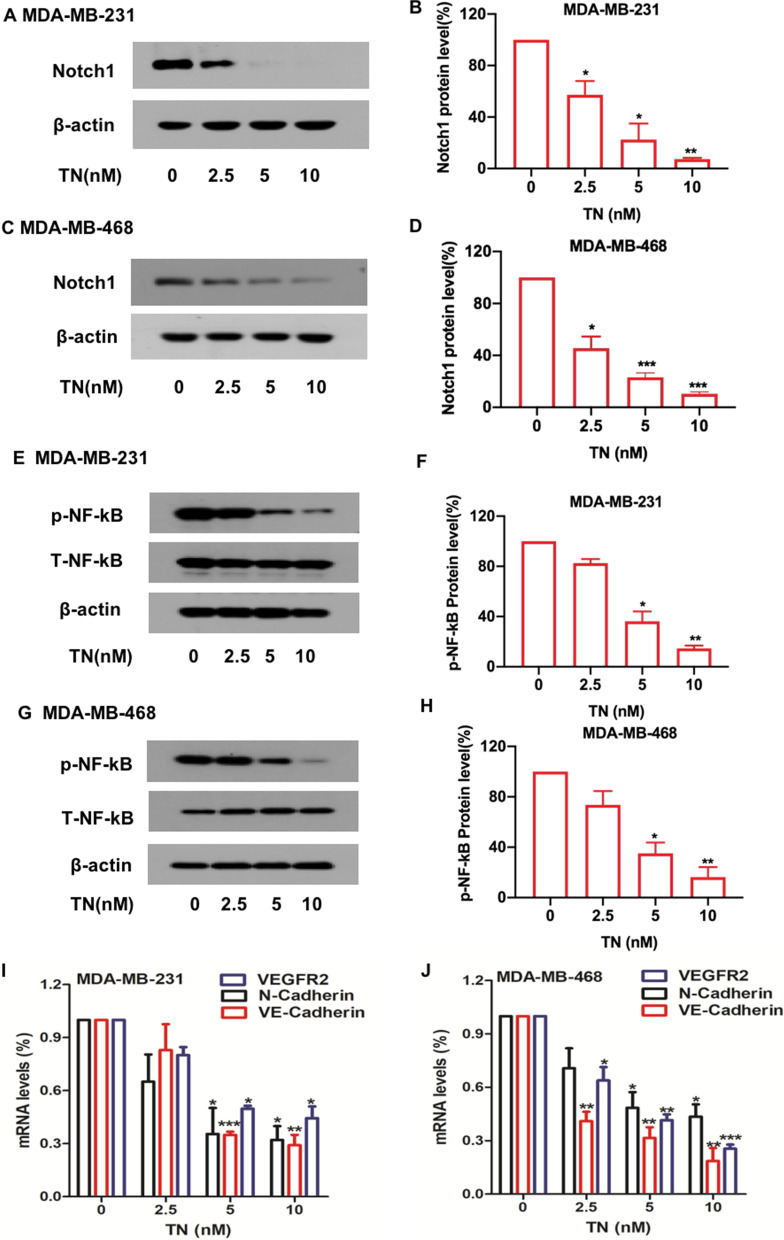
**Triptonide inhibits tumorigenic Notch1 and NF-κB signaling pathways in TNBC cells.** MDA-MB-231 and MDA-MB-468 cells were treated with triptonide (TN) for 72 h at the concentrations of 0–10 nM. Notch1 protein levels were detected by Western blotting (**A**, **C**), and the bands were scanned and statistically analyzed (**B**, **D**). Similarly, TN-mediated reduction of Notch1 protein levels in MDA-MB-468 cells was also verified (**C**, **D**). Additionally, the levels of total NF-κB protein and phosphorylated NF-κB protein (p-NF-κB) were also measured by Western blotting (**E**, **G**), and the bands were scanned and statistically analyzed (**F**, **H**). The mRNA levels of *VE-cadherin, VEGFR2*, and *N-cadherin* in MDA-MB-231 (**I**) and MDA-MB-468 (**J**) cells were assessed by real time quantitative PCR (QT-PCR). The results represent three independent experiments. *P < 0.05, **P < 0.01

We further investigated the effect of triptonide on the levels of other important signaling proteins in TNBC cells. Notably, Notch1 protein levels were markedly reduced in MDA-MB-231 (Fig. 6A, B) and MDA-MB-468 (Fig. 6C, D) cells with triptonide treatment in a concentration-dependent manner. We investigated the effect of triptonide on Notch1 downstream genes and signaling pathways. Triptonide significantly inhibited phosphorylation of NF-κB in MDA-MB-231(Fig. 6E, F) and MDA-MB-468 (Fig. 6G, H) cells in a dose-dependent manner. Additionally, triptonide significantly reduced the expression of tumor angiogenic genes *VE-cadherin* and *VEGFR2* (Fig. 6 I, J) and diminished the levels of the EMT-inducing gene *N-cadherin* in MDA-MB-231 (Fig. 6I) and MDA-MB-468 cells (Fig. 6J). However, Notch1 mRNA levels in these cells were not significantly changed (Additional file 1: Figure S2A–D), suggesting that triptonide triggers Notch1 protein degradation. Triptonide did not significantly change the levels of several other signaling proteins, including p-ERK, p-AKT, p-SRC, p-LYN, p-CREB, p-JAK2, p-STAT3, p-MKK3, p-JNK, PARP, and PTEN (Additional file 1: Figure S2E).

**Fig. 7:**
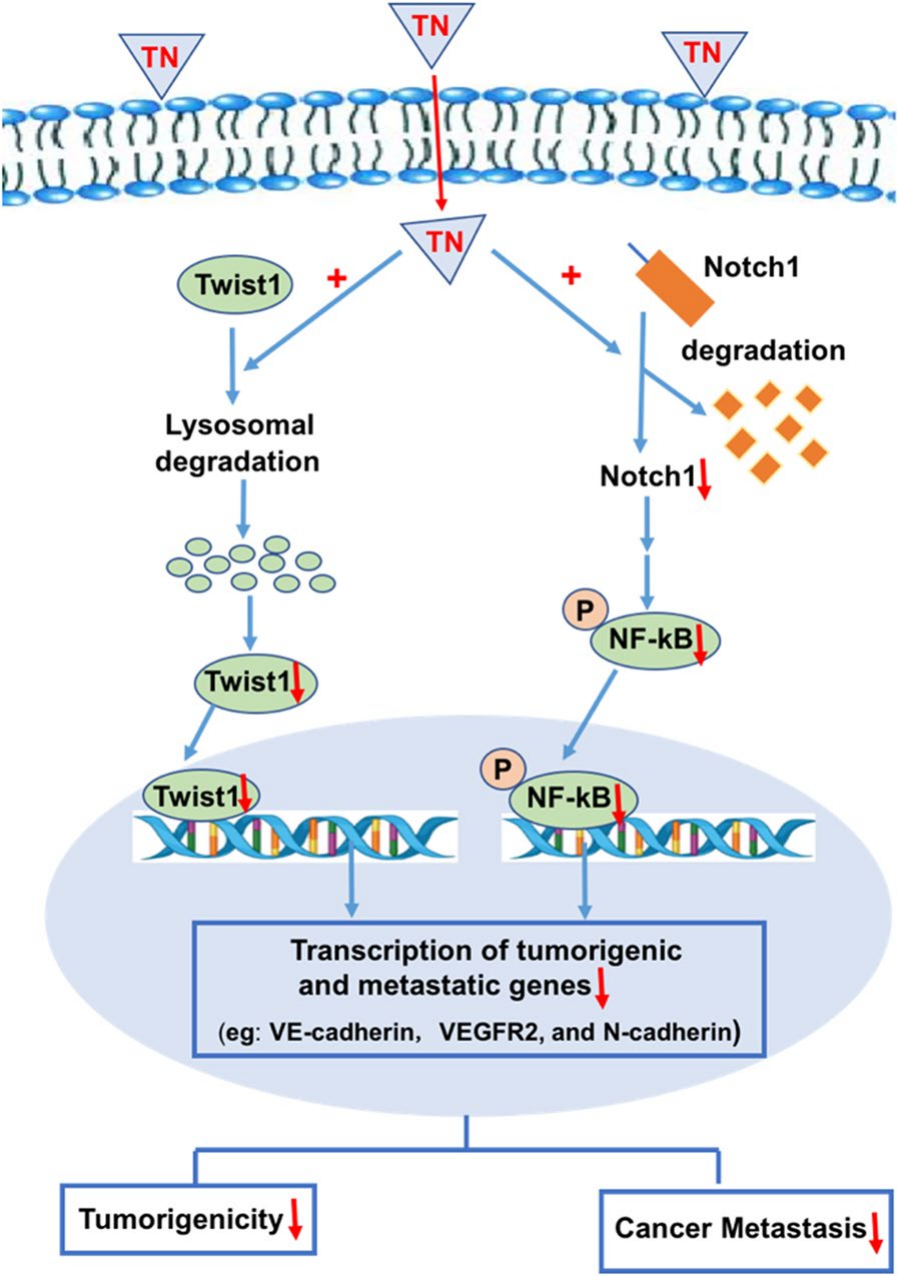
**A schematic summary of triptonide-mediated inhibition of triple-negative breast cancer cell tumorigenesis and metastasis.** Triptonide enters to TNBC cells and triggers the degradation of oncogenic Twist1 and Notch1 proteins, consequently inhibiting their downstream NF-κB signaling, and reducing the expression of angiogenic VE-cadherin and VEGFR2, and EMT-inducing N-cadherin. As a result, triptonide potently suppressed TNBC cell tumorigenesis, migration, invasion, and angiogenesis, tumor growth, and lung metastasis in xenografted mice

Together, these data indicate that triptonide suppresses TNBC cell tumorigenesis and metastasis mainly by triggering the degradation of Twist1 and Notch1 proteins, consequently inhibiting the NF-κB signaling pathway and diminishing the expression of *VE-cadherin*, *VEGFR2*, and *N-cadherin* (Fig. 7).

## Discussion

The initiation and metastasis of TNBC are driven by the overexpression of numerous oncogenic genes and aberrant activation of multiple tumorigenic signaling pathways [6–10]. Despite decades of research, druggable targets for TNBC have been difficult to identify [3, 5, 12, 58], which has hindered the development of effective therapeutics for the disease. In the current study, we explored the ability of an active compound to inhibit TNBC cell tumorigenesis and dissemination in vitro and in vivo. Triptonide triggered the degradation of the Twist1 and Notch1 oncoproteins and inhibited NF-κB signaling and expression of *VE-cadherin*, *VEGFR2*, and *N-cadherin* in TNBC, resulting in potent suppression of TNBC cell tumorigenesis and metastasis. These data suggest that concurrent degradation of the oncoproteins Twist1 and Notch1 and subsequent inhibition of downstream metastatic genes and signaling pathways reverse the aggressive and metastatic phenotypes of TNBC. Our findings provide a new strategy for effectively inhibiting TNBC metastasis and offer a new drug candidate for TNBC treatment.

Twist1 functions as a transcription factor to drive the transcription of numerous oncogenic genes, and the overexpression of Twist1 triggers EMT and cancer metastasis [12, 13, 15, 59]. The reduction of Twist1 expression by thymoquinone [16] and tamoxifen [17] significantly inhibits TNBC tumor growth and metastasis and reverses drug resistance in an in vitro tumor cell model and in mice [16–19]. However, the efficacy of these anti-TNBC agents is generally moderate with no effective TNBC therapeutics in the clinic. In this investigation, we found that 10 nM triptonide nearly eliminates Twist1 protein expression in TNBC cells, which is more potent than other agents reported in the literature [16–19]. Our data indicate that triptonide is a novel, potent Twist1 protein inhibitor, warranting further development of Twist1-targeted drugs for the treatment of TNBC and other types of cancer with aberrant overexpression of Twist1.

Notch1 is a cell surface receptor that plays a pivotal role in supporting tumor cell stemness, EMT, and metastasis [10, 14]. Overexpression of *Notch1* and the activation of the Notch1 signaling pathway are associated with poor prognosis in TNBC patients [24]. However, the efficacy of Notch1-based therapeutics against TNBC has been only moderate and Notch1-targeted drugs are absent in the clinic. In the current study, we found that 10 nM triptonide triggered significant Notch1 degradation in TNBC cells, suggesting that triptonide is a novel and potent Notch1 inhibitor. Whether triptonide directly binds to Twist1 and Notch1 proteins in TNBC and other cancer cells remains to be determined.

Notch1 lies upstream of the transcription factor NF-κB [60], which promotes the expression of numerous angiogenic and metastatic genes [61, 62]. In this study, triptonide significantly inhibited the phosphorylation of NF-κB in TNBC cells, thereby suppressing the expression of numerous oncogenic genes and inhibiting tumorigenesis and cancer metastasis.

Angiogenesis and vasculogenic mimicry play critical roles in tumor growth and cancer metastasis. Several anti-angiogenic drugs, such as Avastin and Sunitinib, have been used in cancer therapy; unfortunately, current anti-angiogenic drugs fail to cure TNBC patients and may actually promote TNBC invasion and metastasis by triggering tumor cell-mediated vasculogenic mimicry [63]. To date, effective drugs that suppress tumor vasculogenic mimicry are absent in the clinic. Triptonide potently suppressed the ability of TNBC cells to form tube-like structures, which is consistent with our recent report that triptonide strongly inhibits vasculogenic mimicry mediated by pancreatic cancer cells [34]. Our mechanistic studies revealed that triptonide suppresses expression of the angiogenic *VE-cadherin* and *VEGFR2* genes, thereby reducing TNBC angiogenesis and vasculogenic mimicry. Our findings provide a new approach for targeting TNBC angiogenesis and vasculogenic mimicry.

As mentioned above, Gao et al. reported that triptonide diminishes TNBC tumor growth via inhibiting expression of several stemness genes, but promoting Snail1 overexpression and induces a Snail-associated feedback mechanism of triptonide-resistance in a single in vitro-produced triptonide-resistant HCC1806 cell line [39]; however, whether triptonide affects Snail1 expression in TNBC cell lines without in vitro artificial induction of triptonide-resistance has not been addressed in the paper [39]. In the current investigation, our data showed that the levels of Snail mRNA and protein in the TNBC cell lines MDA-MB-231 and MDA-MB-468 were not significantly affected by triptonide. These data suggest that the effect of triptonide on Snail1 expression may be limited only in the in vitro produced triptonide-resistant HCC1806 cell line, but is uncommon in TNBC cell lines. Of note, overexpression of the transcription factor Snail drives overexpression of various oncogenic and metastatic genes, activates multiple tumorigenic signaling pathways, and promotes EMT, tumorigenesis, cancer metastasis, and drug resistance [44–53]. Therefore, up-regulation of Snail1 should not be good idea for cancer therapy; instead, suppression of Snail1 should be a sensible strategy for treatment of malignant tumors.

Triptonide exerts anti-cancer effects through down-regulation of various oncogenic genes, including β-catenin, cMyc, Notch1, LXRα, SREBF1 [32–38, 64], particularly, triptonide directly binds to its receptor LXRα, reduces LXRα protein stability in pancreatic cancer cells, and exerts potent anti-cancer effect [32–38, 64]. In the current investigation, we found that triptonide triggers degradation of Twist1 and Notch1 oncoproteins in TNBC cells. Our study reveals a new mechanism of triptonide-mediated tumor suppression.

Triptonide exerts strong anti-cancer effects [32–36, 38, 64] with low toxicity in mice [31, 65], making it an attractive small molecule for TNBC drug development. Furthermore, the combination of triptonide with currently available chemotherapeutics, immunotherapies, and radiotherapy may help shape the future landscape of TNBC treatment.

## Conclusion

Triptonide effectively suppressed the tumorigenesis and metastasis of TNBC via triggering the degradation of Twist1 and Notch1 oncoproteins, consequently downregulating expression of various metastatic and angiogenic genes, and inhibiting NF-κB signaling pathway in TNBC cells. Our findings provide a new strategy for treating highly lethal TNBC and offer a potential novel drug candidate for combatting this aggressive disease.

## Supplementary Information

**Additional file 1: Figure S1.** Triptonide did not significantly change the levels of Snail1 mRNA and protein in the TNBC MDA-MB-231 and MDA-MB-468 cells. **Figure S2**. Triptonide did not significantly affect the levels of Notch1 mRNA and several signaling proteins in TNBC cells.

## Abbreviations

TNBC: Triple-negative breast cancer
EMT: Epithelial–mesenchymal transition
VEGFR2: Vascular endothelial cell growth factor receptor 2
DMEM: Dulbecco's modified Eagle medium
TN: Triptonide
CHX: Cycloheximide
