# Supplementary material for: Triptonide effectively inhibits triple-negative breast cancer metastasis through concurrent degradation of Twist1 and Notch1 oncoproteins
Source: Breast Cancer Res. 2021 Dec 18;23:116. doi: 10.1186/s13058-021-01488-7 (PMC8684143; doi:10.1186/s13058-021-01488-7)
Supplement: Supplementary file 1 — Additional file 1: Figure S1. Triptonide did not significantly change the levels of Snail1 mRNA and protein in the TNBC MDA-MB-231 and MDA-MB-468 cells. Figure S2. Triptonide did not significantly affect the levels of Notch1 mRNA and several signaling proteins in TNBC cells. [file 13058_2021_1488_MOESM1_ESM.docx]

**Supplementary Tables**

**Table 1: The semi-quantitative RT-PCR primers**

| Gene name  (mRNA ID) | Primer name | Primers sequences (5’-3’) | PCR  product  size (bp) |
| --- | --- | --- | --- |
| β-actin  NM_001101.3 | Forw | AAGAGCTACGAGCTGCCTGACG | 420 |
|  | Rev | CGCCTAGAAGCATTTGCGGTGG |  |
| Notch1  NM_017617.5 | Forw | TGCACACTATTCTGCCCCAG | 309 |
|  | Rev | ACTTGAAGGCCTCCGGAATG |  |
| Twist1  NM_000474.4 | Forw | AGCAACAGCGAGGAAGAGCCAGA | 425 |
|  | Rev | AGGAAGTCGATGTACCTGGCCG |  |

**Table 2. The real-time quantitative PCR (QT-PCR) primers**

| Gene name  (mRNA ID) | Primer name | Primers sequences (5’-3’) | PCR product  size (bp) |
| --- | --- | --- | --- |
| β-actin  NM_001101.3 | Forw | CACCATTGGCAATGAGCGGTTCC | 90 |
|  | Rev | GTAGTTTCGTGGATGCCACAGG |  |
| Notch1  NM_017617.5 | Forw | GAGGCGTGGCAGACTATGC | 140 |
|  | Rev | CTTGTACTCCGTCAGCGTGA |  |
| Twist1  NM_000474.4 | Forw | GTCCGCAGTCTTACGAGGAG | 156 |
|  | Rev | GCTTGAGGGTCTGAATCTTGCT |  |
| Snail1  NP_005976.2 | \| Forw \| \| --- \| \| Rev \| | ACTGCAACAAGGAATACCTCAG  GCACTGGTACTTCTTGACATCTG | 242 |

**Supplementary Figures**


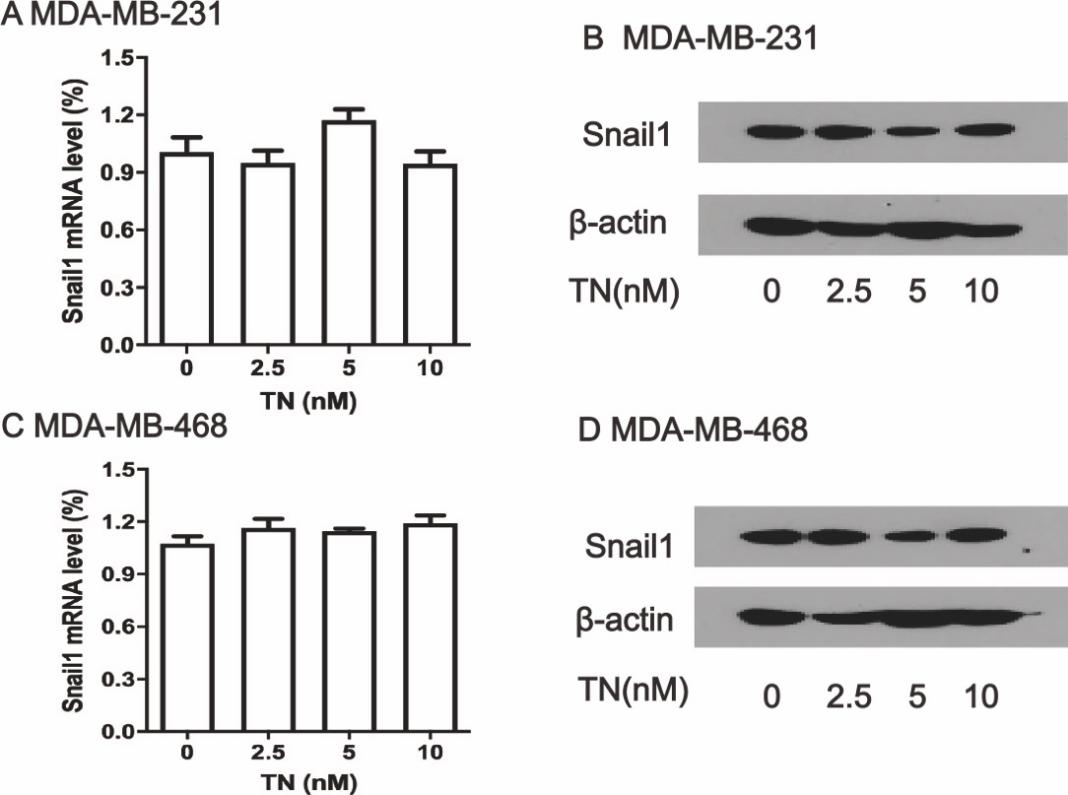


**Supplementary Figure S1. Triptonide did not significantly change the levels of Snail1 mRNA and protein in the TNBC MDA-MB-231 and MDA-MB-468 cells.**

After MDA-MB-231 and MDA-MB-468 cells were treated with triptonide (TN) at the concentrations of 0-10 nM for 72 h, the levels of Snail1 mRNA were measured by QT-PCR (A, C) and Snail1protein were detected by Western blotting (B, D), respectively. Triptonide do not significantly affect Snail1 expression in TNBC cells. The results represent three independent experiments.


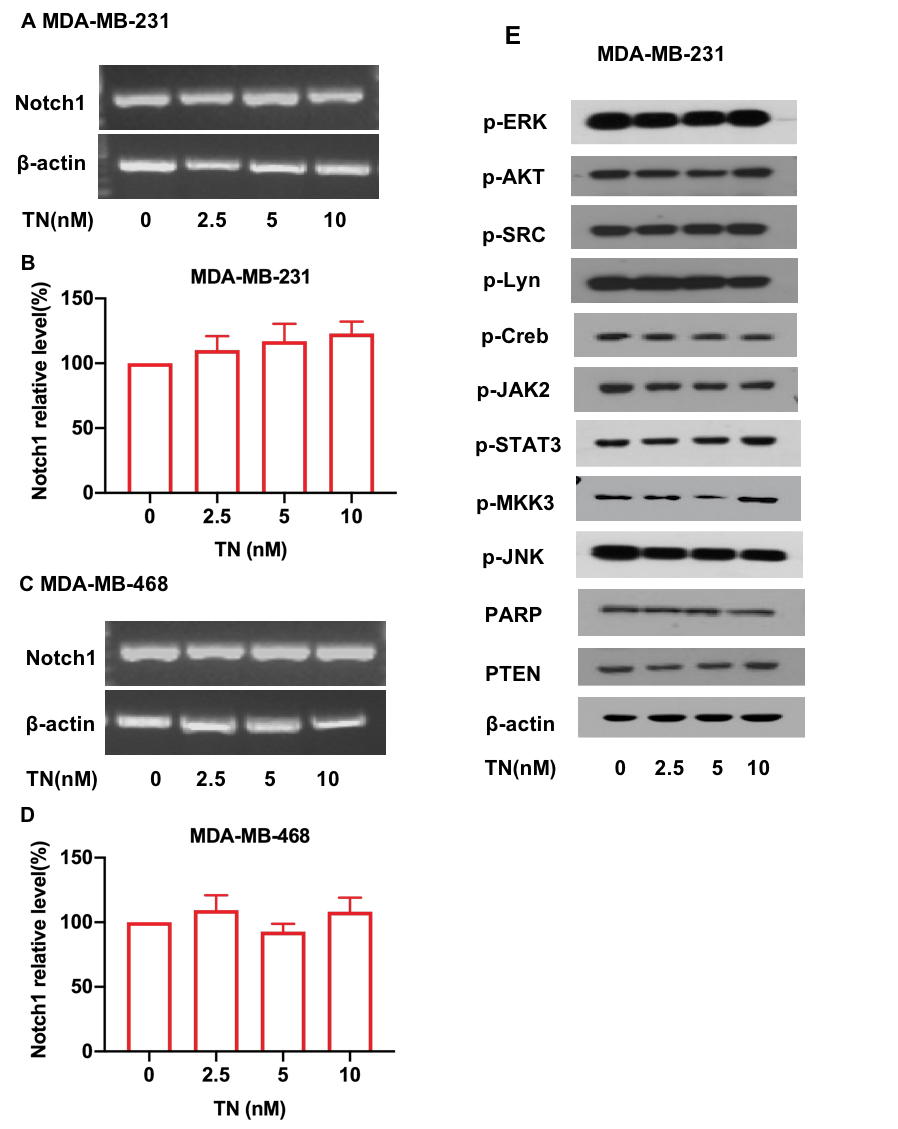


**Supplementary Figure S2.** **Triptonide did not significantly affect the levels of Notch1 mRNA and several signaling proteins in TNBC cells.**

Notch1 mRNA levels in MDA-MB-231 and MDA-MB-468 cells were quantitated by RT-PCR (A, C) and QT-PCR (B, D) after treatment by triptonide (TN) at the concentrations of 0-10 nM. The levels of several signaling proteins p-ERK, p-AKT, p-SRC, p-LYN, p-CREB, p-JAK2, p-STAT3, p-MKK3, p-JNK, PARP, and PTEN, were detected by Western blotting (E). The results represent three independent experiments.
